# Aging yeast gain a competitive advantage on non‐optimal carbon sources

**DOI:** 10.1111/acel.12582

**Published:** 2017-03-01

**Authors:** Stephen Frenk, Grazia Pizza, Rachael V. Walker, Jonathan Houseley

**Affiliations:** ^1^ Epigenetics ProgrammeBabraham InstituteCambridgeCB22 3ATUK; ^2^ Flow Cytometry Core FacilityBabraham InstituteCambridgeCB22 3ATUK; ^3^ Present address: Department of GeneticsUniversity of North CarolinaChapel HillNC27599USA

**Keywords:** yeast, aging, specialist, generalist, evolution of aging

## Abstract

Animals, plants and fungi undergo an aging process with remarkable physiological and molecular similarities, suggesting that aging has long been a fact of life for eukaryotes and one to which our unicellular ancestors were subject. Key biochemical pathways that impact longevity evolved prior to multicellularity, and the interactions between these pathways and the aging process therefore emerged in ancient single‐celled eukaryotes. Nevertheless, we do not fully understand how aging impacts the fitness of unicellular organisms, and whether such cells gain a benefit from modulating rather than simply suppressing the aging process. We hypothesized that age‐related loss of fitness in single‐celled eukaryotes may be counterbalanced, partly or wholly, by a transition from a specialist to a generalist life‐history strategy that enhances adaptability to other environments. We tested this hypothesis in budding yeast using competition assays and found that while young cells are more successful in glucose, highly aged cells outcompete young cells on other carbon sources such as galactose. This occurs because aged yeast divide faster than young cells in galactose, reversing the normal association between age and fitness. The impact of aging on single‐celled organisms is therefore complex and may be regulated in ways that anticipate changing nutrient availability. We propose that pathways connecting nutrient availability with aging arose in unicellular eukaryotes to capitalize on age‐linked diversity in growth strategy and that individual cells in higher eukaryotes may similarly diversify during aging to the detriment of the organism as a whole.

The progressive decline commonly associated with aging results in loss of fitness and eventually of viability. However, the almost universal conservation of aging amongst eukaryotes indicates that aging existed in the single‐celled ancestors of extant eukaryotes (Jones *et al*., 2014). Therefore, aging emerged not in multicellular animals but in single‐celled organisms and it remains to be tested whether age‐related physiological changes always reduce the fitness of individual cells. If the fitness impact of aging differs between single‐celled and multicellular organisms, it is possible that aging or age‐modulatory pathways are under positive selection. Formidable arguments refute this concept in higher eukaryotes, but pathways that evolved under historical positive selection may still modulate aging in higher eukaryotes albeit not necessarily to a useful end.

Fitness is a measure of the transfer of genes to the next generation and, in non‐social organisms, is largely determined by number of offspring. Fecundity is a function of both individual and environment, and there can be trade‐offs between life‐history strategies with fitness returns not always being equal under different environments. We asked whether age‐linked physiological changes in single‐celled eukaryotes represent a loss of specialization that may enhance adaptation to alternate environments (Fig. 1A). To this end, we designed a competitive growth assay for young and aged budding yeast in static environments and during environmental change (Fig. 1B). *Saccharomyces cerevisiae* is a glucose specialist, but can efficiently metabolize other carbon sources such as galactose in the absence of glucose.

**Figure 1:**
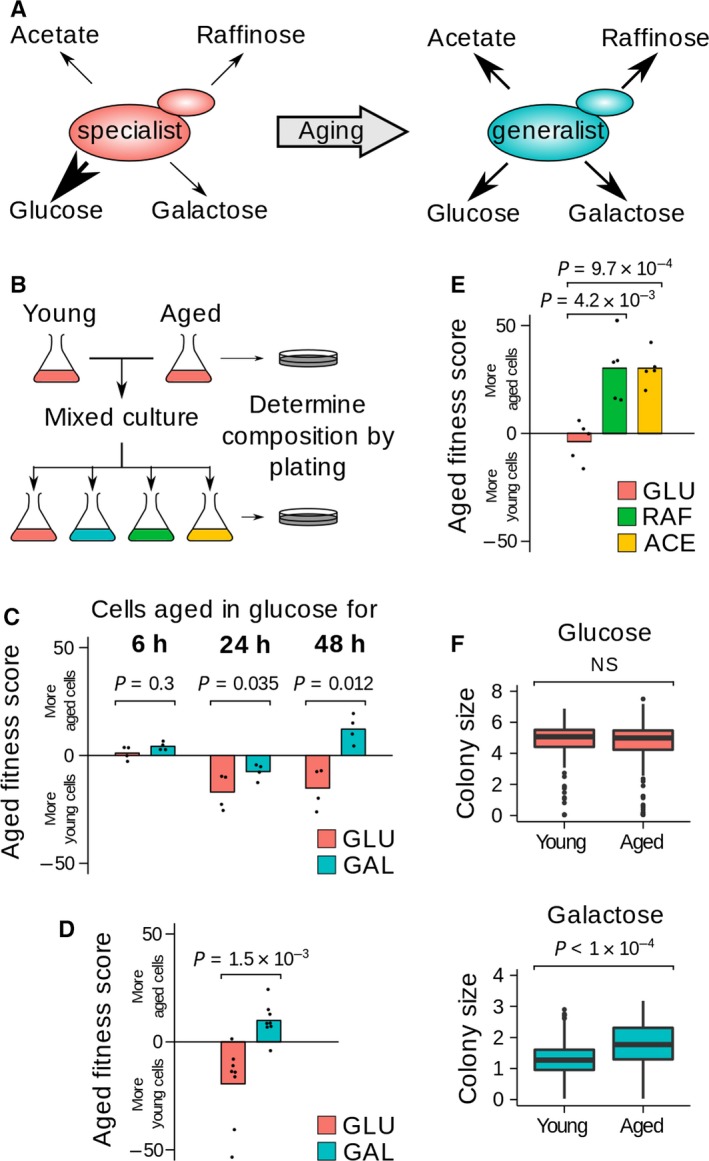
Analysis of fitness in young and aged cells. (A) Age‐linked specialist to generalist transition. (B) Competition assay: isogenic cells of different ages with different antibiotic resistance markers are mixed, and the composition of the mixture determined before and after outgrowth in different media by spreading on antibiotic plates. (C) Competition of log‐phase cells versus cells aged in glucose for 6, 24 or 48 h with outgrowth in glucose and galactose. Analysis by paired *t*‐test, *n* = 4, see Table S1 (Supporting information) for age distributions. (D) Competition between cells aged for 6 and 48 h in glucose as in (C), *n* = 8. (E) Competition between cells grown in glucose for 6 and 48 h then outgrown in glucose, raffinose or acetate as in (C), analysis by one‐way anova,* n* = 5. (F) Size distributions of colonies on glucose or galactose plates formed by cells aged for 2 or 18 h (~1 or ~11 divisions). ~300 viable cells plated per condition, analysis by *t*‐test.

We employed the mother enrichment programme (MEP) (Lindstrom & Gottschling, 2009) to test the fitness of replicatively aged yeast (Fig. S1, Supporting information). We competed young cells against cells aged for 6, 24 and 48 h (Table S1, Supporting information, gives age distributions for each time); at 6 h, the aged cells are fully viable, reproductive viability starts to decline at 24 h and median lifespan has passed at 48 h (Lindstrom & Gottschling, 2009; Fig. S2, Supporting information). Cells of different ages were mixed, inoculated in glucose or galactose and outgrown to saturation (Fig. 1B). A change in the proportion of cells derived from the young and aged populations between inoculation and saturation indicates a fitness difference in that specific condition and is expressed as an Aged Fitness Score, with positive scores indicating that older cells engendered more progeny than younger cells during outgrowth (Eqn S1, Supporting information).

Consistent with an age‐related physiological decline, young cells outcompeted 24‐ and 48‐h aged cells when aging and outgrowth were performed in glucose, whereas young and 6‐h aged cells showed similar fitness (Fig. 1C GLU). However, when cells were aged in glucose but outgrown in galactose, the fitness advantage of young cells relative to 24‐h aged cells was reduced and strikingly 48‐h aged cells outcompeted young cells in every replicate (Fig. 1C GAL, *n* = 4). This shows that the relationship between age and fitness depends on the environment.

The MEP system is not activated in young cells, and young populations also contain 50% newborn cells with an extended cell cycle (Hartwell & Unger, 1977). As both factors may confound competition assays, we compared cells aged for 6 and 48 h. Again, aged cells outcompeted young cells on galactose in all replicates (Fig. 1D, *n* = 8), and also on raffinose or acetate (Fig. 1E). Populations of aged cells only show a significant advantage at 48 h by which time the average age is 16–30 generations, and such cells are rare in the wild. However, individual cells may prosper much earlier. We measured colonies formed on glucose or galactose plates by cells aged for 2 or 18 h (~1 or ~11 generations) and observed that aged cells form significantly larger colonies on galactose, demonstrating a significant growth advantage even at intermediate age (Figs 1F and S3, Supporting information).

Time‐course and redilution experiments provided no evidence for permanent genetic adaptation to galactose (Figs 2A and S4, Supporting information); instead, aged cells have a transient advantage rapidly lost in progeny. This could be explained either by aged cells growing more rapidly than young cells on galactose or by aged cells resuming growth more rapidly after an environmental change. To distinguish these, we compared cells aged in galactose prior to outgrowth in glucose or galactose (Fig. 2B). As before, aged cells outcompeted young cells in galactose showing that an environmental change is not required. Furthermore, we did not observe an accelerated galactose response in aged cells or a dependence on nutrient storage (Fig. S5, Supporting information).

**Figure 2:**
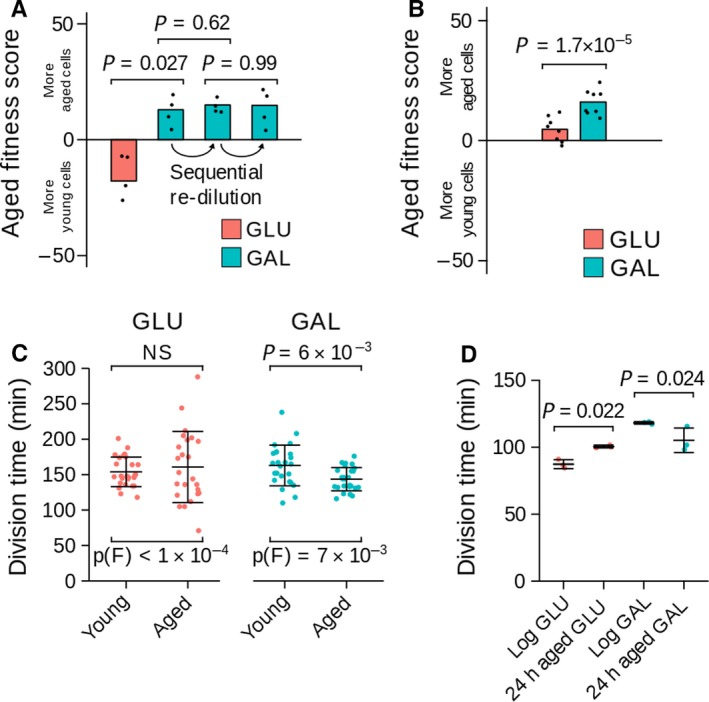
Aged cells show enhanced growth in galactose. (A) Sequential 1:25 000 redilution in galactose of cells from 48‐h competition in Fig. 1C, analysis by one‐way anova,* n* = 4. (B) Competition between 6 and 48 h aged cells as in (D), but with pregrowth and aging in galactose, *n* = 8. (C) Cell division time in young and aged wild‐type cells aged for 1 or ~13–14 divisions by micromanipulation on glucose or galactose plates. Analysis by unpaired *t*‐test with Welch's correction, *P* values – comparison of means; *P*(F) – *P* values from *F*‐test comparing variances. (D) Average cell division time obtained by OD measurement for log‐phase cells, or by counting bud scars after 24 h growth for aged cells. Analysis by one‐way anova,* n* = 3.

This implies that aging cells divide faster than young cells in galactose. We measured cell cycle times on glucose and galactose of wild‐type (non‐MEP) diploid cells aged for ~14 generations by micromanipulation (cf. 16–30 generations for competition assays), and also of the daughters of these cells (Figs 2C and S6, Supporting information). On glucose, the cell division times of mothers and daughters were similar, although division time heterogeneity increased with age as reported (Liu *et al*., 2015). On galactose the opposite was observed: division time decreased significantly with age while heterogeneity lessened. To independently confirm this remarkable observation, we measured average cell division time across 24‐h aging by bud scar counting and again observed that aged cells divide faster than young cells on galactose (Fig. 2D).

Our experiments show that aging does not entail a simple decline in fitness for yeast. Rather, aging cells lose glucose specialization as gene expression studies have suggested (Lin *et al*., 2001; Lesur & Campbell, 2004), but gain fitness for other carbon sources. This is very surprising, but is consistent with conflicts between optimal life‐history strategies evident from mutants with reduced fitness on glucose but improved fitness in other media (Qian *et al*., 2012). Our data show that aging in yeast marks a transition between life‐history strategies appropriate for different environments. This transition may emerge serendipitously from age‐linked physiological changes, or may represent a defined programme that has evolved to be associated with (but is not necessarily causal to) the aging process. Either way, it creates a selective pressure for the evolution of aging regulatory systems as aged cells would be under positive selection in non‐glucose and fluctuating environments. Consequently, as nutrient responsive signalling pathways evolved in early eukaryotes, manipulation of the aging process may have provided a way to tune growth strategy for current and future nutrient availability, and indeed aging is accelerated by galactose (Liu *et al*., 2015). To what extent such regulatory systems have been conserved in higher eukaryotes remains to be determined. However, loss of specialization seems intimately connected to aging, and the inability of cells to perform specialized functions is a major contributor to aging pathology in higher eukaryotes. It is therefore tempting to speculate that the modulation of aging by nutrient signalling in multicellular organisms stems from an ancient mechanism to control specialization.

## Funding

Funding from Wellcome Trust [088335 and 110216], EpiGeneSys Network of Excellence, BBSRC and MRC.

## Conflict of interest

The authors declare that they have no conflict of interests.

## Supporting information


**Appendix S1.** Materials and methods.
**Fig. S1** Schematic of the MEP system as used for competition assays.
**Fig. S2** Viability curve of competition strains in YPD with 1 µm β‐estradiol.
**Fig. S3** Reproducible growth advantage on galactose at intermediate age.
**Fig. S4** Time‐course of Aged Fitness Score after glucose to galactose shift.
**Fig. S5** Aged cells do not show accelerated *GAL* gene induction.
**Fig. S6** Cell division times of daughters from young or old mothers.
**Table S1** Age distributions of young and aged cultures.
**Table S2** Yeast strains used in this research.
**Table S3** Oligonucleotides used in this research.
**Equation S1.** Calculation of Aged Fitness Score.
