# Supplementary material for: Aging yeast gain a competitive advantage on non‐optimal carbon sources
Source: Aging Cell. 2017 Mar 1;16(3):602–4. doi: 10.1111/acel.12582 (PMC5418195; doi:10.1111/acel.12582)
Supplement: Supplementary file 1 — Appendix S1. Materials and methods. Fig. S1 Schematic of the MEP system as used for competition assays. Fig. S2 Viability curve of competition strains in YPD with 1 µm β‐estradiol. Fig. S3 Reproducible growth advantage on galactose at intermediate age. Fig. S4 Time‐course of Aged Fitness Score after glucose to galactose shift. Fig. S5 Aged cells do not show accelerated GAL gene induction. Fig. S6 Cell division times of daughters from young or old mothers. Table S1 Age distributions of young and aged cultures. Table S2 Yeast strains used in this research. Table S3 Oligonucleotides used in this research. Equation S1. Calculation of Aged Fitness Score. [file ACEL-16-602-s001.docx]

**Ageing yeast gain a competitive advantage on non-optimal carbon sources**

Stephen Frenk, Grazia Pizza, Rachael V. Walker and Jonathan Houseley

**Materials and Methods**

*Yeast Strains and Culture Conditions*

Yeast strains containing the Mother Enrichment Program (MEP) constructs (Lindstrom & Gottschling 2009) were obtained from Dan Gottschling. The KAN strains were created by transforming these MEP haploids with the KanMX6 cassette from the pFA6a-KanMX6 plasmid (Longtine *et al.* 1998), resulting in replacement of the NatMX6 cassette by KanMX6. The insert was amplified from pFA6a-KanMX6 using oligonucleotides pFA6a F1 *Sac*I and pFA6a R1 (Table S3). Haploids were then mated to produce the diploids used for the competition assay – SF129 (KAN) and SF132 (NAT) (Table S2), and ploidy was verified by flow cytometry. The original MEP diploid UCC5185 (Lindstrom & Gottschling 2009) was used for colony size assays and bud scar counting, while a diploid created by mating S288C-derived haploids BY4741 and BY4742 (EuroSCARF) was used for micromanipulation. Other strains were derived by standard methods. Strains are listed in Table S2, and oligonucleotides used for transformation in Table S3.

Cells were grown in YP media (1% yeast extract, 2% peptone, 2% sugar). Media components were purchased from Formedium. For competition assays, YP media was autoclaved prior to addition of sugar from a filtered 20% stock, while for colony size and bud scar assays media was prepared by filtration without autoclaving. Liquid cultures were grown at 30°C with shaking at 200 rpm. For micromanipulation, plates were prepared by autoclaving YP containing 2% agar before addition of sugar to 2% from a filtered 20% stock immediately before pouring.

*Design of the competition assay*

Highly aged cells represent a tiny proportion of a yeast population – cells at the median lifespan are less than 1 in 10 million. Furthermore, in a competition assay designed to measure fitness across environmental change, it is critical that the aged cells are not subject to any stressful purification procedure. The “Mother Enrichment Program” (MEP) (Lindstrom & Gottschling 2009) fulfils both of these criteria: MEP cells are engineered such that in the presence of estradiol all new-born daughter cells are rendered permanently inviable, but cells born after estradiol removal can divide as normal. Therefore, growth in estradiol yields a population highly enriched for aged cells, and when the population is removed from estradiol only the aged cells are viable. This means that MEP populations can be used directly in competition assays, requiring only a wash with media to remove estradiol (Figure S1).

Cells for competition assays should be as genetically identical as possible, but also easily distinguished. To achieve this, we replaced a nourseothricin (*NAT*) marker already present in MEP cells with a *KAN* gene conferring resistance to G418. Neither antibiotic marker affects cell growth under normal conditions, but *NAT*-marked and *KAN*-marked cells (and their progeny) can be distinguished by spreading on plates containing nourseothricin or G418, allowing the composition of cell mixtures to be readily determined. To ensure that both strains acted equivalently, all experiments were performed in pairs where one test used young *NAT* and aged *KAN* cells while the other used young *KAN* and aged *NAT* cells.

In each competition experiment, one strain was aged for a specific amount of time by the addition of β-estradiol. The other strain was maintained in log phase for the same amount of time. The two strains were then mixed together in equal proportions, with a compensation for the progressive decline in viability with age calculated using the graph in Figure S2 (50% at six hours, 40% at 24 hours and 15% at 48 hours). A sample of the mixture was spread on G418 and on nourseothricin plates and the rest of the mixture was aliquoted into outgrowth cultures. When the outgrowth cultures had reached saturation, a sample of each culture was spread on G418 and on nourseothricin plates. The result of the competition was expressed as an “aged fitness score”, which was calculated by subtracting the percentage of cells in the inoculation mixture belonging to the aged strain from the percentage of cells in the final outgrowth culture that were descended from the aged strain (Equation S1).

*Competition assay method*

For aged versus log competition assays with ageing in glucose, competition assay strains containing the *KAN* or *NAT* gene (hereafter referred to as the “K” and “N” strains respectively) were streaked onto YPD plates and incubated for two to three days until single colonies appeared. Single colonies from each of the two strains were picked and used to inoculate separate 4 ml YPD cultures. The cultures were incubated overnight then diluted into fresh YPD and grown overnight again, ensuring that the cells would be in mid log phase the following day. In the afternoon, these cultures were used to inoculate ageing and log YPD cultures. The ageing cultures were inoculated at a cell density of 2 x 10^4^ cells/ml in YPD with 1 µM β-estradiol and 1 µg/ml ampicillin and incubated for the specified ageing time while the log cultures were maintained in log phase for the same amount of time by daily dilution. Pairs of cultures were mixed together to create two aged/log mixtures: one containing aged K cells and log N cells and the other containing aged N cells and log K cells. The number of cells in the mixture belonging to each strain was 2 x 10^4^ x (n+1), where n was the number of outgrowth cultures. An appropriate volume of aged culture was added to compensate for the expected percentage of viable cells (50% at six hours, 40% at 24 hours and 15% at 48 hours). The mixture was centrifuged and washed with YP (no carbon source) then re-suspended in 200 µl YP per outgrowth culture plus an extra 200 µl YP. 200 µl aliquots of the mixture were used to inoculate 25 ml outgrowth cultures (so that each culture was inoculated with 4 x 10^4^ cells). The remaining mixture (~200 µl) was diluted with 7.8 ml YP and 50 µl aliquots of this dilution were spread onto KAN and NAT plates (cells were plated in triplicate). Plates were incubated for two to three days until colonies were clearly visible. The outgrowth cultures were grown to saturation. 0.5 µl was taken from each outgrowth culture and diluted with 10 ml YP. 50 µl of this dilution was plated onto KAN and NAT plates. Plates were incubated for two to three days until colonies were clearly visible. Plates from the mixture and outgrowth cultures were imaged with the plate in a consistent location in the image and colonies were counted using ImageJ (v1.50i) with this macro:

makeOval(228, 66, 928, 912);

run("Clear Outside");

run("16-bit");

setAutoThreshold("Default dark");

run("Convert to Mask");

run("Watershed");

run("Analyze Particles...", "circularity=0.50-1.00 show=Outlines display clear summarize");

All downstream analysis was performed using custom R scripts. The ratio of colony counts between the KAN and NAT plates was used to determine the percentage of aged cells in each mixture/outgrowth culture. Aged Fitness Scores were calculated by subtracting the percentage of aged cells in the initial mixture from the percentage of aged cells in the outgrowth culture.

For competitions in which 48-hour aged cells were competed against 6-hour aged cells, the procedure was identical to that outlined above with the exception that an ageing culture was set up six hours prior to the competition (creation of the log/aged mixtures) and used in place of the log culture.

*YPGal re-dilution experiment*

The saturated galactose outgrowth cultures of a 48 hour versus log phase competition were used to inoculate a 25 ml YPGal culture at a dilution of 1 in 25,000. These cultures were grown to saturation and re-diluted again in YPGal at the same dilution then grown to saturation. Aged Fitness Scores were calculated from cells spread on antibiotic plates as described above.

*Competition time course*

A 48 hour-aged versus six hour-aged competition assay with ageing in glucose and outgrowth in glucose and galactose was carried out as described above up until creation of the aged/log mixtures. 12.5 ml outgrowth cultures were inoculated with 8 x 10^4^ cells from the aged/log mixtures. Immediately after addition of the mixture, 200 µl of each outgrowth culture was plated onto KAN and NAT plates to record the 0 hour time point. The outgrowth cultures were incubated and cells were plated at three, six, 24 and 48 hours after inoculation. At the three hour time point, 200 µl of cells from each culture was plated. At the six hour time point, 100 µl of cells from each culture was plated. At the 24 hour time point, 0.5 µl of glucose outgrowth culture was diluted into 10 ml YP, 1 µl of galactose outgrowth culture was diluted into 1 ml YP and 50 µl of each dilution was plated onto KAN and NAT plates. At the 48 hour time point, 0.5 µl from each outgrowth was diluted into 10 ml YP and plated (both cultures had reached saturation by this time point).

*Colony size analysis*

6 replicate cultures of MEP cells were grown to mid-log phase in YPD, diluted to 2x10^4^ cells/ml and incubated 18 hours with 1µM β-estradiol. 6 other cultures were diluted 1:5,000 in YPD from the same log phase cells and grown overnight to OD ~0.1-0.2, before dilution to 1x10^6^ cells/ml and induction for 2 hours with 1µM β-estradiol. These were timed so that 2 and 18 hour incubations finished simultaneously. 1 ml of each culture was harvested by centrifugation, washed with 1 ml YPD, re-suspended in 1ml YPD, then 125 µl (18 hour cultures) or 2.5 µl (2 hour cultures) was diluted to 1 ml with YPD. 40µl of this dilution was plated each on YPD and YPGal plates which were then incubated at 30°C for exactly the same time period (of ~2 days). Colony density was low (~50 per plate) to minimize potential for growth interference, and merged colonies were removed based on circularity. Plates were imaged and colony sizes determined using ImageJ (v1.50i) with the following macro:

makeOval(228, 66, 928, 912);

run("Clear Outside");

run("16-bit");

setAutoThreshold("Default dark");

run("Convert to Mask");

run("Analyze Particles...", "size=2-Infinity pixel circularity=0.92-1.00 show=Outlines display clear");

*Bud scar analysis*

MEP cells were grown to mid-log phase in YPD, then 0.1x10^7^ cells were washed twice with PBS, labelled with 100µl 3mg/ml Sulfo-NHS-LC-Biotin (Pierce 10538723) in PBS for 30 min at room temperature, washed with PBS and inoculated in 50 ml YPD with 1 µM β-estradiol and 5 µg/ml ampicillin. Cells were grown for 24 hours at 30°C, then harvested by centrifugation and fixed in 70% ethanol. Cells were rehydrated in PBSE (PBS+ 2mM EDTA) then loaded on a 1 ml Percoll gradient (77% percoll (Sigma), 160 mM NaCl, centrifuged 15 min at 20,000 *g*) and centrifuged 4 min at 2,000 *g*. Cells at the interphase were discarded, while cells in the gradient were recovered by addition of 1ml PBSE and centrifugation for 1 min at 2,000 *g*. Cells were re-suspended in 0.5 ml PBSE with 12.5µl streptavidin magnetic beads (Miltenyi Biotec) and incubated 5 min at room temperature on a wheel before applying to a pre-equilibrated LS column (Miltenyi Biotec). After binding cells were washed with 8 ml PBSE and eluted in 1 ml PBSE, then pelleted by centrisfugation 1 min at 20,000 *g*. Cells were stained with 0.3µl 1mg/ml Alexa-594 streptavidin (Thermo Fisher) and 0.6µl 1mg/ml Alexa-488 WGA (Thermo Fisher) in 300µl PBS / 5% normal goat serum / 0.3% PBS for 30 min at room temperature, washed with PBS then mounted in VectaShield (VectorLabs). Bud scars were counted using a 100x objective on an Olympus BX40, 10 cells were counted per replicate and the identity of the samples was blinded for the experimenter to avoid counting bias. The average bud scar counts minus 1.83 (which is the average age of a logarithmic population with age 0 cells removed, these cells are rendered inviable on β-estradiol addition) was calculated, giving the number of divisions in 24 hours. The division time was calculated as 24 x 60 divided by the number of divisions. Log phase doubling times were obtained by measuring OD_600_ of a log phase culture of the same cells on the same media starting at OD 0.1 (1x10^6^ cells/ml).

*qRT-PCR*

MEP cells were labelled, aged for 48 hours and purified as for bud scar analysis. RNA was extracted using the GTC:phenol method (described in (Cruz & Houseley 2014)), reverse transcribed using SuperScript II after DNase I treatment and subjected to qPCR analysis using the primers listed in Table S3.

*Imaging flow cytometry*

7 x 10^6^ cells were incubated in 1 ml PBS with 6 µl 1 mg/ml Alexa Fluor^®^-647 conjugated wheat germ agglutinin for one hour at room temperature with gentle shaking. Cells were washed and resuspended in 1 ml flow run buffer (200 mM Tris-HCl pH 7.5, 20 mM EDTA) then analysed using an ImageStream Mark II imaging cytometer (Merck Millipore Amnis). Data was analysed using Amnis Ideas software.

For Gal1-GFP induction measurements, MEP cells homozygous for a *GAL1-eGFP* fusion were labelled, aged for 48 hours and purified as described for bud scar analysis above. Cells were then stained with Alexa Fluor^®^-647 conjugated wheat germ agglutinin and imaged as above

*Micromanipulation*

Aged cell measurements: SF92 cells were re-streaked on YPD or YPGal plates and incubated at 30°C for 3 days , then a single colony was re-streaked on a fresh plate and incubated for 1 hour at 30°C. Using an MSM400 micromanipulator (Singer), 40 mother cells with small buds were transferred to marked locations on the plate. Cells were examined every hour and any daughter cells removed and recorded. The plate was incubated at 30°C during the day and 4°C at night wrapped in parafilm. Cells were aged for an average of 13 generations (glucose) or 14 generations (galactose), some cells were discarded because they ceased to divide or mothers and daughters could not be separated. On the 5th day, plates were removed from the fridge, equilibrated for 1 hour at room temperature and any daughters removed and discarded. Cells were then examined every 10-15 minutes and the following times noted: time at which first bud is just visible, time at which second bud is just visible (this interval is the mother’s cell division time given in Fig. 2C), time at which daughter cell grown from the first bud shows a bud (the interval between the mother’s first bud and daughter’s first bud is the daughter’s cell division time given in Fig. S6). During the 5th day, plates were maintained at room temperature (22 ± 1 °C) to avoid temperature fluctuations of shifting to 30° between observations, explaining the difference between division times measured in this experiment and division times measured at 30°.

For measurements in young cells, we aimed to recreate the conditions experienced by ageing cells as accurately as possible: we therefore allowed cells to divide at low density followed by overnight incubation in the fridge prior to measurement. Cells were streaked on YPD or YPGal plates at 30°C for 24 hours, then stored at 4°C overnight. After equilibration of the plates at room temperature for 1 hour, 30 mothers with small buds were transferred to marked locations. Once the cells divided, the mothers were discarded and daughter cells examined every 10-15 minutes as above, recording the same events.

**References**

Cruz C, Houseley J (2014). Endogenous RNA interference is driven by copy number. *eLife*. **3**, e01581.

Hartwell LH, Unger MW (1977). Unequal division in Saccharomyces cerevisiae and its implications for the control of cell division. *The Journal of cell biology*. **75**, 422-435.

Lindstrom DL, Gottschling DE (2009). The mother enrichment program: a genetic system for facile replicative life span analysis in Saccharomyces cerevisiae. *Genetics*. **183**, 413-422, 411SI-413SI.

Longtine MS, McKenzie A, 3rd, Demarini DJ, Shah NG, Wach A, Brachat A, Philippsen P, Pringle JR (1998). Additional modules for versatile and economical PCR-based gene deletion and modification in Saccharomyces cerevisiae. *Yeast*. **14**, 953-961.

**Supplemental Figures, Tables and Equation**


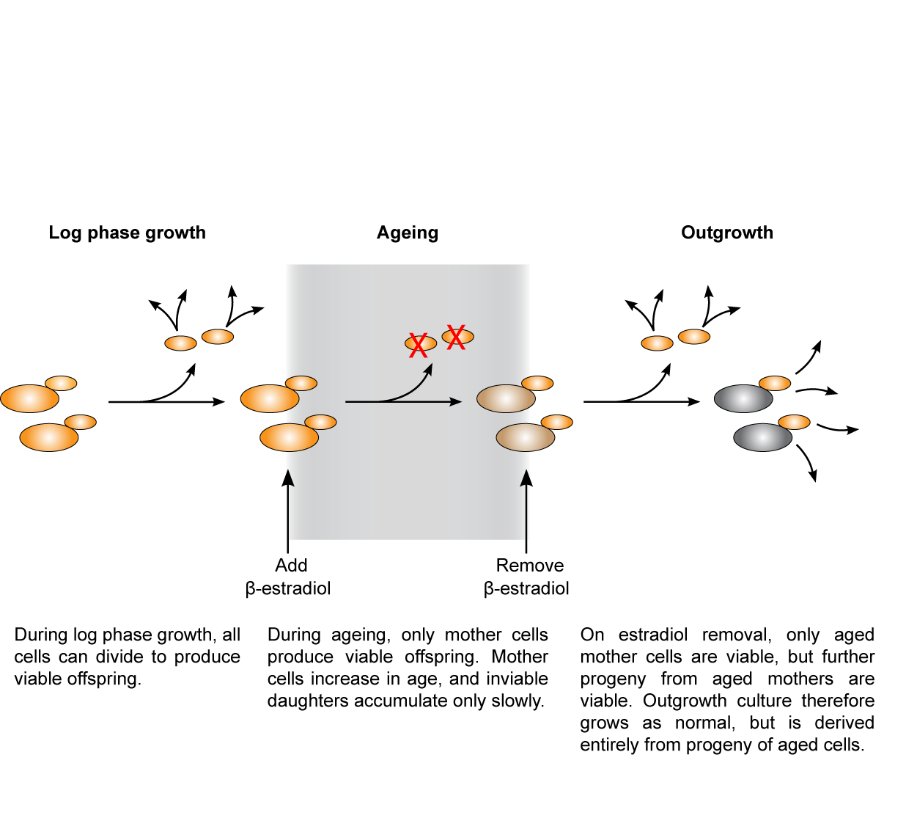


**Figure S1: Schematic of the MEP system as used for competition assays**

*
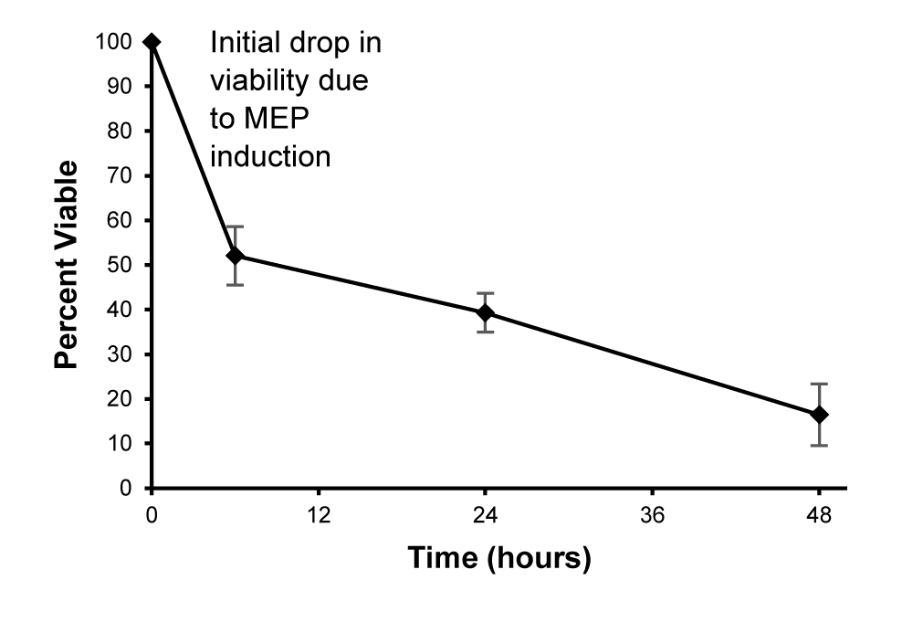
*

**Figure S2: Viability curve of competition strains in YPD with 1 µM β-estradiol**

Viability was determined by harvesting samples at the indicated times, washing and plating cells on YPD followed by colony counting. Mean and standard deviation of five independent cultures is shown. Measured changes in viability are a combination of the effect of the MEP system and the effect of ageing. When estradiol is added to a culture to initiate the MEP system, the 50% of cells that are new-born in any log phase culture are immediately rendered inviable, explaining the drop in viability from 0-6 hours. Further loss of viability is due to replicative ageing, which causes 20% of the remaining cells to lose viability at 24 hours and 70% at 48 hours.

**
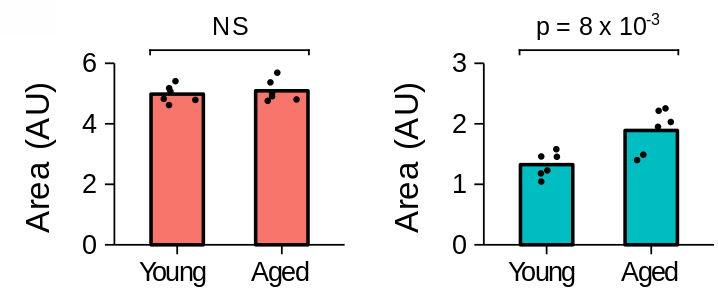
**

**Figure S3. Reproducible growth advantage on galactose at intermediate age**

Six individual cultures each of cells aged for 2 or 18 hours (~1 or ~11 divisions by bud scar counting) in YPD were plated on YPD (red) or YPGal (blue) plates at low density (~50 viable cells/plate). Colony sizes were determined after 2 days incubation at 30°. Each point represents the median colony size from a single culture (~50 cells). Sizes are in arbitrary units (pixels). n=6 for each condition, p values calculated by *t* test. The sizes of colonies produced by aged cells plated on YPGal is significantly greater than those formed by young cells on YPGal plates, showing higher fitness of many cells in the aged population even at this intermediate age. The size data from all the plates of each class was pooled together to generate the distributions shown in Fig. 1F.

**
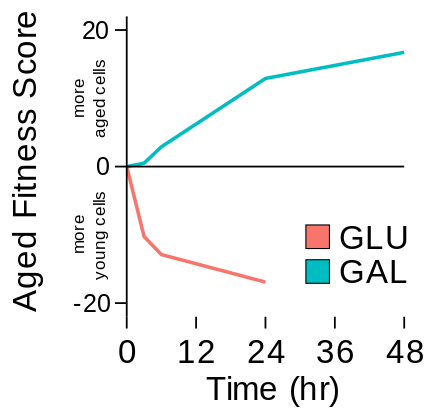
**

**Figure S4: Time-course of Aged Fitness Score after glucose to galactose shift**

To determine when during outgrowth the change in Aged Fitness Score is accrued, we performed a time course measuring Aged Fitness Score at 3, 6, 24 and 48 hours after inoculation in glucose or galactose. Both young and aged cells showed a pause in growth after the shift to galactose (0-3hr) but the aged cells then divided earlier than the young cells causing a rapid increase in Aged Fitness Score from 3-6 hours. After this point, at which both young and old populations had doubled at least once, the rate of change in Aged Fitness Score progressively decreased with time. This is consistent with new-born cells inheriting no growth advantage from the aged parents. Similarly, in glucose the young cells divided much more rapidly at the start of the competition but the rate of change in Aged Fitness Score decreased with time, consistent with the progeny of old and young cells sharing equivalent growth rates. This time-course compares Aged Fitness Scores starting with six-hour and 48-hour aged cells grown in glucose and outgrown in glucose or galactose, showing an average of two experiments at each point.

**
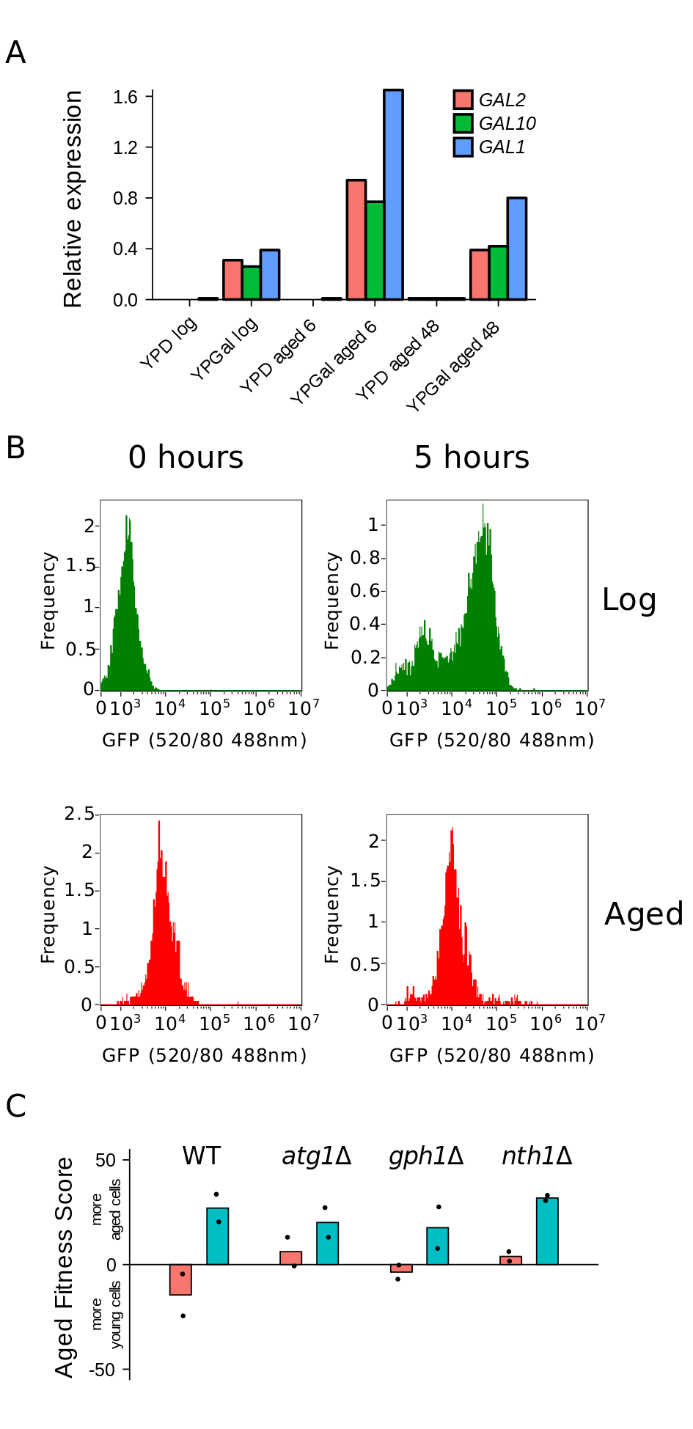
**

**Figure S5: Aged cells do not show accelerated *GAL* gene induction**

**A**: qRT-PCR analysis of mRNA from *GAL2, GAL10* and *GAL1* in log phase cells and cells aged for 6 or 48 hours. Cells were grown in YPD, and where indicated were harvested by centrifugation and re-suspended in YPGal for 3 hours at 30° prior to purification. Expression levels were normalized to *ACT1* mRNA. **B**: Imaging cytometry analysis of Gal1-eGFP in cells grown to log-phase or aged for 48 hours in YPD (0 hours), and then harvested by centrifugation and re-suspended in YPGal for 5 hours at 30° (5 hours). The increased fluorescence in aged vs. log 0 hour samples was also observed in untagged controls (not shown) and therefore represents autofluorescence. Gal1-GFP induction after 5 hours was minimal in the aged cells but strong in the log phase cells. **C**: Results of 6 vs. 48hr competition assays in wild-type, *atg1*Δ, *gph1*Δ and *nth1*Δ mutants. These mutants are deficient in autophagy, glycogen utilization or trehalose utilization respectively.


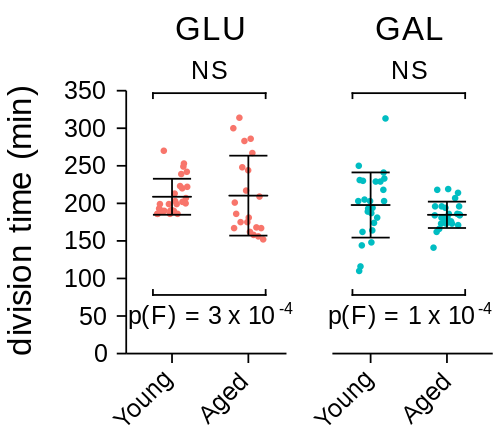


**Figure S6: Cell division times of daughters from young or old mothers**

For the micromanipulation experiment shown in Fig. 2C, the time taken for the first division by each daughter cell was also measured. This was taken as the time interval between mother producing a bud and that bud giving rise to a budded daughter cell. This time is uniformly longer than division time of the mother cell as previously noted (Hartwell & Unger 1977). Although the division times between conditions vary little, the division time on glucose for daughters of aged cells is more heterogeneous than for those of young cells, whereas the converse is true for cells grown on galactose. Analysis by unpaired *t*-test with Welch’s correction, p values for difference in mean are not significant, p(F) – p value derived from F test for difference in variance.

| **Age category** | **Distribution** |
| --- | --- |
| Young cells (log phase) | 44% age 0 (new born), 24% age 1, 15% age 2, 9% age 3, 4% age 4, 2% age 5, 1% age 6, 1% age 7 and above.  All cells are replicatively viable. |
| 6 hour aged | 6% age 0, 14% age 1, 17% age 2, 19% age 3, 16% age 4, 11% age 5, 7% age 6, 4% age 7, 3% age 8, 2% age 9, 1% age 10 and above.  All aged cells are replicatively viable, but 50% of the starting cells which were new-born on addition of estradiol to the culture at t=0 have been rendered inviable. Therefore measured replicative viability is 50%. |
| 24hr aged | heterogeneous population primarily age 6-20, few age 0 (based on data in Lindstrom and Gottschling, 2009).  Measured replicative viability is at 40% since 20% of the aged cells loose viability between 6 and 24 hours. |
| 48hr aged | heterogeneous population primarily age 16-30, no age 0 (based on data in Lindstrom and Gottschling, 2009).  Measured replicative viability is at 15% since 70% of the aged cells loose viability between 6 and 48 hours. |

**Table S1:** Age distributions of young and aged cultures. For log phase cells and six hour-aged cells, age distributions were empirically determined by bud scar counting using imaging flow cytometry. The age distributions for 24 and 48 hours are based on previously published data for this strain (Lindstrom and Gottschling, 2009). Replicative viability data is from Figure S2.

JH935 MEP diploid UCC5185 from Gottschling lab (Lindstrom & Gottschling 2009)

*ade2::hisG his3 leu2 lys2/+ met15Δ::ADE2/+ ura3Δ0 trp1Δ63 hoD::SCW11pr-Cre-EBD78-NatMX loxP-UBC9-loxP-LEU2 loxP-CDC20-Intron-loxP-HPHMX*

SF132 MEP NAT diploid re-mated and ploidy verified by flow cytometry

*ade2::hisG his3 leu2 lys2/+ met15Δ::ADE2/+ ura3Δ0 trp1Δ63 hoD::SCW11pr-Cre-EBD78-NatMX loxP-UBC9-loxP-LEU2 loxP-CDC20-Intron-loxP-HPHMX*

SF129 MEP KAN diploid mated and ploidy verified by flow cytometry

*ade2::hisG his3 leu2 lys2/+ met15Δ::ADE2/+ ura3Δ0 trp1Δ63 hoD::SCW11pr-Cre-EBD78-KanMX loxP-UBC9-loxP-LEU2 loxP-CDC20-Intron-loxP-HPHMX*

SF92 BY4741xBY4742 diploid

leu2Δ0 met15Δ0/+ lys2Δ0/+ ura3Δ0 his3Δ1

SF141 MEP *GAL1-eGFP* diploid

*ade2::hisG his3 leu2 lys2/+ met15Δ::ADE2/+ ura3Δ0 trp1Δ63 hoD::SCW11pr-Cre-EBD78-NatMX loxP-UBC9-loxP-LEU2 loxP-CDC20-Intron-loxP-HPHMX GAL1-eGFP-HisMX6*

JH1118 MEP *atg1*Δ NAT diploid

*ade2::hisG his3 leu2 lys2/+ met15Δ::ADE2/+ ura3Δ0 trp1Δ63 hoD::SCW11pr-Cre-EBD78-NatMX loxP-UBC9-loxP-LEU2 loxP-CDC20-Intron-loxP-HPHMX atg1::TRP1*

JH1123 MEP *atg1*Δ KAN diploid

*ade2::hisG his3 leu2 lys2/+ met15Δ::ADE2/+ ura3Δ0 trp1Δ63 hoD::SCW11pr-Cre-EBD78-KanMX loxP-UBC9-loxP-LEU2 loxP-CDC20-Intron-loxP-HPHMX atg1::TRP1*

SF161 MEP *gph1*Δ NAT diploid

*ade2::hisG his3 leu2 lys2/+ met15Δ::ADE2/+ ura3Δ0 trp1Δ63 hoD::SCW11pr-Cre-EBD78-NatMX loxP-UBC9-loxP-LEU2 loxP-CDC20-Intron-loxP-HPHMX gph1::TRP1*

SF160 MEP *gph1*Δ KAN diploid

*ade2::hisG his3 leu2 lys2/+ met15Δ::ADE2/+ ura3Δ0 trp1Δ63 hoD::SCW11pr-Cre-EBD78-KanMX loxP-UBC9-loxP-LEU2 loxP-CDC20-Intron-loxP-HPHMX gph1::TRP1*

SF168 MEP *nth1*Δ NAT diploid

*ade2::hisG his3 leu2 lys2/+ met15Δ::ADE2/+ ura3Δ0 trp1Δ63 hoD::SCW11pr-Cre-EBD78-NatMX loxP-UBC9-loxP-LEU2 loxP-CDC20-Intron-loxP-HPHMX nth1::TRP1*

SF167 MEP *nth1*Δ KAN diploid

*ade2::hisG his3 leu2 lys2/+ met15Δ::ADE2/+ ura3Δ0 trp1Δ63 hoD::SCW11pr-Cre-EBD78-KanMX loxP-UBC9-loxP-LEU2 loxP-CDC20-Intron-loxP-HPHMX nth1::TRP1*

**Table S2**: Yeast strains used in this research

*Strain construction*

pFA6a F1: GAATTCGAGCTCGTTTAAAC

pFA6a R1 *Sac*I: GTCAGCATGTGAGCTC CGGATCCCCGGGTTAATTAAC

GAL1 UP45 2: ATCATCGTCTCTAAACCAGCATTGGGCAGCTGTCTATATGAATTA CGG ATC CCC GGG TTA ATT AAC

GAL1 DN45 2: AAAAATGAGAAGTTGTTCTGAACAAAGTAAAAAAAAGAAGTATAC GAATTCGAGCTCGTTTAAAC

ATG1 UP45 1: ATATTTTCAAATCTCTTTTACAACACCAGACGAGAAATTAAGAAACGGATCCCCGGGTTAATTAAG

ATG1 DN45 1: TAGCAGGTCATTTGTACTTAATAAGAAAACCATATTATGCATCACGAATTCGAGCTCGTTTAAAC

GPH1 UP: TACAATTTCAGTAAAGCAGCTTTCAACTTAATTTAATTTTGAACACGGATCCCCGGGTTAATTAAG

GPH1 DN: TAGAAATGGGGAGAAGGGTGCCAAAGAGATAAAAAAAACTCCCAGGAATTCGAGCTCGTTTAAAC

NTH1 UP: GCCTGATAAACAAAAAAAGAAAAATTAACAAAAAAAATCAGTAGACGGATCCCCGGGTTAATTAAG

NTH1 DN: GCCTGATAAACAAAAAAAGAAAAATTAACAAAAAAAATCAGTAGACGGATCCCCGGGTTAATTAAG

*qPCR*

ACT1 1 F: TTTCCATATCGTCCCAGTTG

ACT1 1 R: TCCTACGTTGGTGATGAAGC

GAL2 2 F: CATCGCCGTCTTATGTCCTA

GAL2 2 R: CAAGAAACTAGTGTGCCTCTCAA

GAL10 3 F: TGCCCGTAACTTTGTATGGA

GAL10 3 R: AGGTTTGTGTCGTGAGTGGA

GAL1 3 F: ATTACGGTCGTTGCAGAACA

GAL1 3 R: ATTACGGTCGTTGCAGAACA

**Table S3**: Oligonucleotides used in this research

Aged Fitness Score

% progeny from aged cells at saturation

% progeny from aged cells at inoculation

=

-

**Equation S1:** Calculation of Aged Fitness Score.
